# Rapid diagnostics for SARS-CoV-2 virus: point-of-care testing and lessons learned during the pandemic

**DOI:** 10.4155/bio-2021-0100

**Published:** 2021-07-21

**Authors:** Trieu Nguyen, Anders Wolff

**Affiliations:** ^1^Department of Biotechnology & Biomedicine, Technical University of Denmark, Ørsteds Plads, DK-2800 Kgs, Lyngby, Denmark

**Keywords:** coronavirus, COVID-19, *in vitro* diagnostic regulation, lessons, nucleic amplification, pandemic, point-of-care testing, rapid diagnostics, reverse-transcription polymerase chain reaction, SARS-CoV-2

The COVID-19 pandemic originated from an outbreak of novel coronavirus in Wuhan City, in the Hubei Province of China, in late 2019. The virus was later named severe acute respiratory syndrome-related coronavirus 2, or SARS-CoV-2, in February 2020 [1]. More than a year into the pandemic (at the time we write this article in May 2021), the world is still struggling with testing, isolating clusters, curfews and lockdowns as there is a lack of an efficient antiviral for SARS-CoV-2 and the distribution of COVID-19 vaccines is still a work in progress. The pandemic is currently in its third wave. This commentary article focuses on early, onsite and rapid detection, such as point-of-care (POC) diagnostics of the SARS-CoV-2. POC serology tests, namely antibodies-related tests, are not discussed in this viewpoint as they are not applicable for early detection of SARS-CoV-2 due to the antibody's long-overdue presentation response (which can commence at 2 weeks or later) in the majority of cases after exhibiting symptoms.

## Test, test, test: from PCR testing to the POC testing

*“Test, test, test. Test every suspected case,”* Tedros Adhanom Ghebreyesus, the World Health Organisation (WHO) Director-General, pressed at a news conference in Geneva, Switzerland in March 2020. Testing is certainly essential to isolate clusters and prevent the spread of SARS-CoV-2 and its new variants. The current standard diagnostic method, namely the real-time reverse-transcription polymerase chain reaction (RT-rPCR), is incompetent for widespread testing as it endures from prolonged turn-around times (>24 h to deliver a result [2]) and primarily counts on high biosafety-level laboratories and skillful technicians. In Denmark, a COVID-19 RT-rPCR test certificate can be issued within 24 h. On the other hand, Denmark has also implemented rapid POC antigen test for SARS-CoV-2 virus and an antigen test result can be issued within an hour after the sample was taken.

POC tests have the probability to tremendously improve healthcare in many ways, enabling not only rapid detection but also mitigation of the spreading of the disease, especially in remote areas. POC testing means the test is performed at or near the patient and conducted by an untrained operator [3,4]. This concept is not new and can be traced back to the 15th century when urine was inspected at the patient's home [5]. POC testing is especially useful for screening and rapid testing in places such as at the airports.

When it comes to clinical testing, namely disease diagnostics, besides the time factor, there is also the criteria of sensitivity and specificity of the analytical techniques. Both antigen POC testing and nucleic amplification POC testing have high specificity with >95% [6]. The latter, however, owns higher sensitivity [7,8] mainly due to the high concentration of the genetic materials after the amplification. However, the amplification reaction and sample preparation of the nucleic amplification POC testing contributed to the longer turn-around time compared with antigen POC testing.

## SARS-CoV-2 POC testing: state of the art & lessons

The achievement of rapid testing for SARS-CoV-2 has been incredible for the antigen test [6] as the results of such tests (with a nasopharyngeal swab) can be delivered within 15 min after the sample is taken. One example is that in the EU, Denmark has enforced mandatory onsite antigen tests at the Copenhagen (CPH) airport to all passengers who arrive during the entry restriction from early January to the time of writing this article. The antigen test results are obtained quickly onsite within 15 min after a technician takes the samples, and all the tests are free of charge in Denmark.

The commercial antigen test is, however, costly. For example, in April 2021, one antigen test and a certificate of the test for SARS-CoV-2 can cost approximately 200 euros in Finland [9].

The antigen test is, however, less sensitive (>80% sensitivity) compared with a nucleic acid amplification test (e.g., a PCR or a LAMP test with >90% sensitivity) [7,8].

The commercialization of isothermal nucleic acid amplification-based POC diagnostic device for SARS-CoV-2 has so far come from established companies (that is a company-based POC device and not a laboratory-based device originated from a university) such as Abbott (ID NOW platform) and Cuehealth (Cue Covid-19 test) [10].

Until now (at the time of writing this article), a university-based POC device using isothermal amplification for SARS-CoV-2, such as real-time reverse transcription loop-mediated isothermal amplification, has not yet been successfully commercialized to feed into the market's urgent need and serve the pandemic.

There are many reasons that may have tumbled the commercialization process; among them, the following three can be considered the most significant:

(i) The test's sensitivity for clinical samples is a hurdle even though it can be overcome in the future;
(ii) The cooperation between University laboratories and clinical facilities for testing actual samples is still limited due to the short-handed workforce in the middle of the pandemic;
(iii) Commercialization of the *in vitro* diagnostic medical devices (IVDs) needs to meet the standard regulation such as ISO (14971:2019, 23640:2015 and so on), the European Directive 98/79/EC (IVDD) and *in vitro* Diagnostic Regulation (IVDR). These regulations have to be taken into account simultaneously with the laboratory research investigation, in the beginning, to meet the safety regulation and quality demands nationally and internationally, such as the emergency use authorization.

## Conclusion & future perspective

Testing is one of the central measures to mitigate the Covid-19 pandemic and prevent the potential outbreaks of the SARS-CoV-2 variants. The more viruses replicate, the more they mutate. The more they mutate, more dominant variants will appear, which could emerge and pose severe health and economic problems to our community. At the time we write this manuscript, a new variant, namely B.1.617, of SARS-CoV-2 has emerged and detected in India [11]. COVID-19 cases in India surge with more than 300,000 positive cases recorded daily – the highest number that has been recorded daily up till now in a country. The best thing we can do to prevent these circumstances, in general, is to eliminate the circulating of the virus. For that, one of the best tools to implement is rapid diagnostics and isolate clusters using POC testing. Further, the commercialization and emergency use authorization need to account simultaneously with laboratory research investigations at the first instance.
